# Aberrant expression and regulatory network of splicing factor-SRSF3 in tumors

**DOI:** 10.7150/jca.42645

**Published:** 2020-03-15

**Authors:** Yingying Che, Lin Fu

**Affiliations:** Institute of Chronic Disease, Qingdao Municipal Hospital, Qingdao University, Qingdao 266000, China.

**Keywords:** SRSF3, tumor, alternative splicing, biological functions

## Abstract

Alternative splicing facilitates the splicing of precursor RNA into different isoforms. Alternatively spliced transcripts often exhibit antagonistic functions or differential temporal or spatial expression patterns. There is increasing evidence that alternative splicing, especially by the serine-arginine rich (SR) protein family, leads to abnormal expression patterns and is closely related to the development of cancer. SRSF3, also known as SRp20, is a splicing factor. Through alternative splicing, it plays important roles in regulating various biological functions, such as cell cycle, cell proliferation, migration and invasion, under pathological and physiological conditions. Deregulation of SRSF3 is an essential feature of cancers. SRSF3 is also considered a candidate therapeutic target. Therefore, the involvement of abnormal splicing in tumorigenesis and the regulation of splicing factors deserve further analysis and discussion. Here, we summarize the function of SRSF3-regulated alternative transcripts in cancer cell biology at different stages of tumor development and the regulation of SRSF3 in tumorigenesis.

## Introduction

Posttranscriptional modification is an essential component of the genetic code of eukaryotic genomes. Alternative splicing is an important process involved in posttranscriptional modification 1. It is known that ~95% of human genes can be spliced 2. Through this process, the noncoding introns are removed, and coding exons containing the correct translational information are left to maintain the stability of eukaryotic organisms. Alternative splicing plays important roles in cell proliferation and differentiation, and thus participates in multiple diseases, including cancer 3, 4.

Alternative splicing refers to the process by which different splicing isomers are produced from a pre-mRNA by different splicing methods, leading to final protein products with different phenotypes, different functional characteristics or antagonistic effects on tumors 5. Alternative splicing is accomplished via the spliceosome. The spliceosome is mainly composed of five kinds of small nuclear ribonucleic proteins (snRNPs) and three kinds of non-snRNPs 6-8. Among them, non-snRNPs include the hnRNP protein family, serine/arginine-rich splicing factor (SR) protein family and SR- related protein family. SR protein can bind to pre-mRNA and influence the interaction between snRNP splicing factors and pre-mRNA, thus promoting or inhibiting the recognition and utilization of splicing sites 9.

At present, SRSF1-12 (also named SC35, SRp20, SRp75, SRp40, SRp55, 9G8, SRp46, SRp30c, SRp38, NET2, SRrp35) have been extensively studied 10. These SR proteins are involved in many intracellular physiological functions, such as mRNA translocation, mRNA stability, genomic stability and mRNA translation 11-14. Abnormal expression of splicing factors has been known to interfere with normal splicing of pre-mRNA, resulting in abnormal diseases and cancer 15, 16.

**Figure 1:**
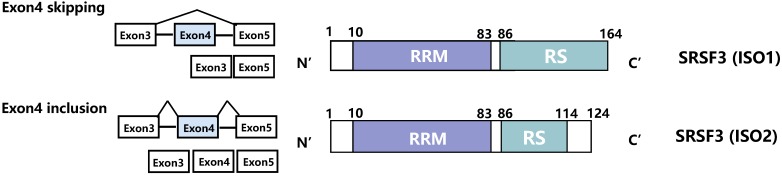
** Schematic represents the isoforms and domains of the SRSF3.** The full-length SRSF3 contains an RRM (10-83bp) domain and an RS domain (86-164bp). The RRM domain is used to recognize and bind pre-mRNA, while the RS domain plays a splicing role. The SRSF3 exon 4 skipping promotes the production of full-length SRSF3 (ISO1). The SRSF3 exon 4 inclusion leads to the generation of truncated SRSF3 (ISO2). This splicing is regulated by SRSF1, HnRNP L, SLU7, PTBPs, RBM4 and circSMARCA5.

Serine/arginine-rich splicing factor 3 (SRSF3) is the smallest member of the SR protein family. SRSF3 is relatively conserved and binds to specific pre-mRNAs. It contains one RRM (RNA receptor binding domain) and one RS domain (Figure 1). The RRM domain, located at the N-terminus, can specifically recognize and bind to pre-mRNA. Thus, it is the key factor by which SR proteins determine the splicing site 17. The RRM domain can interact with pre-mRNA cis-elements in a concentration-dependent manner and then regulate the alternative splicing of these genes 18, 19. For example, exogenous noncoding SRSF3 (exon 4 inclusion) can increase the expression of endogenous SRSF3 and p53 in a dose-dependent way 20. In addition, the high concentration of SR proteins can even replace U1 snRNP and restore splicing activity to pre-mRNA 21, 22. The RS domain, located at the C-terminus, is composed of alternately arranged arginine and serine, which mainly mediate the interaction between protein and mRNA or protein and protein. Through interaction, the SR protein can bind to other splicing factors and aggregate near splicing sites of pre-mRNA. Therefore, the RS domain determines the splicing activity of the SR protein and is also a necessary region for splicing pre-mRNA 23.

## SRSF3-regulated alternative splicing in tumors

Overexpression of SRSF3 in cancer cell lines has been found through database analysis and tissue sample detection. SRSF3 is highly expressed in many tumor tissues, such as the thyroid, ovary, lung, colon, breast, stomach, skin and bladder 15, 24-26. Thus, the expression of SRSF3 might be a useful biomarker for diagnosis and prognosis.

**Figure 2:**
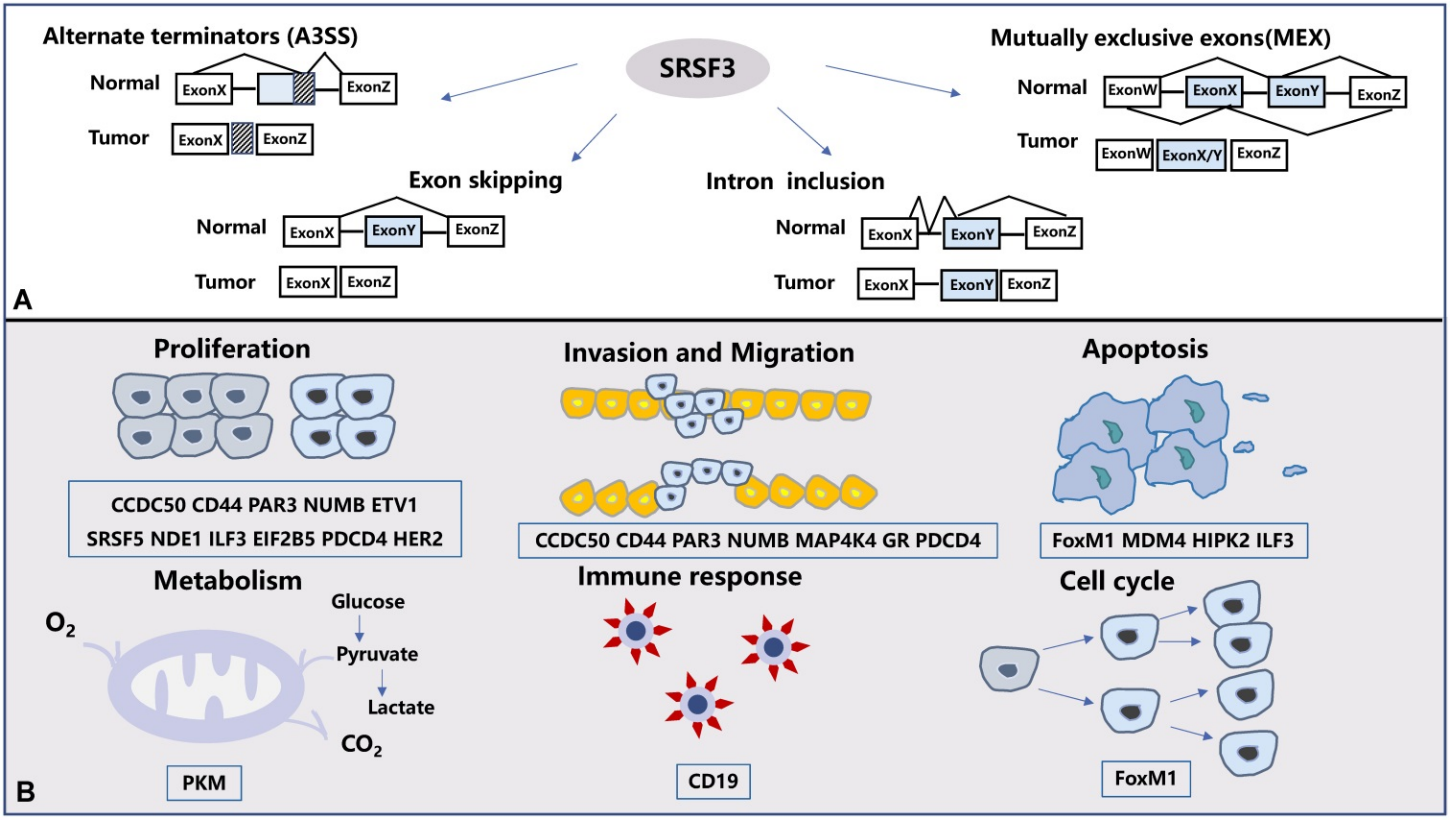
** The alternative splicing and biological functions regulated by SRSF3**. (A). SRSF3 is involved in four kinds of splicing ways in human tumors: exon skipping, intron retention, alternate terminators(A3SS) and mutually exclusive exons (MEX). (B). SRSF3-mediated alternative splicing results in different protein isoforms that regulate a variety of biological processes in tumors. The biological functions regulated by SRSF3 includes tumor proliferation, metastasis, apoptosis, cell metabolism, immune response and cell cycle.

At present, there are five main types of alternative splicing that have been discovered: exon skip (ES), retained intron (RI), alternative promoters (A5SS), alternative terminators (A3SS), and mutually exclusive exons (MEX) 27. Among them, SRSF3 is involved in four types of splicing (Figure 2A).

### SRSF3 and exon skipping (ES)

#### SRSF3 promotes exon skipping

CCDC50 (long isoform including alternative exon 6, CCDC50L) has been recognized as a tyrosine- phosphorylated protein. Overexpression of SRSF3 can promote CCDC50 exon 6 skipping to form CCDC50S (short isoform lacking alternative exon 6, CCDC50S) (Table 1). Compared with CCDC50L, CCDC50S has a higher expression and tissue specificity in hepatocellular carcinoma (HCC). SRSF3 can also improve the stability of CCDC50S in the cytoplasm and further increase the activation of the Ras/Foxo4 signaling pathway. Further, upregulated expression of CCDC50S can increase the proliferation and metastasis of HCC 28. CCDC50S can be used as a new therapeutic target and prognostic biomarker for HCC.

SRSF3 can also mediate some receptors. CD44, an important membrane receptor of hyaluronic acid (HA), is also involved in various processes, including tumor proliferation and metastasis 29-31. Overexpression of SRSF3 promotes skipping of CD44 exons 12-14 to form the CD44E variant, which enhances tumorigenesis (Table 1) 32.

TAR-DNA binding protein (TARDBP, also named TDP43) belongs to the hnRNP family 33. The expression of SRSF3 is positively correlated with TDP43 in Triple-negative breast cancer (TNBC). In particular, TDP43 interacts with SRSF3, and they can enhance exon12 skipping of PAR3 (Δ12PAR3 isoform) and NUMB (Δ12NUMB isoform). In contrast to PAR3 and NUMB, which inhibit the proliferation and metastasis of tumors, Δ12PAR3 and Δ12NUMB are known as oncogenes 34-36. Therefore, overexpression of Δ12PAR3 and Δ12NUMB can rescue the inhibition caused by TDP43 or SRSF3 knockdown in TNBC (Table 1) 37.

#### SRSF3 inhibits exon skipping

In addition to exon skipping, SRSF3 can also promote the exon inclusion (Table 1). Transcription factor ETS variant 1 (ETV1) is an oncogene belonging to the E twenty-six (ETS) family that regulates cell proliferation, differentiation, migration and angiogenesis 38. ETV1-FL regulates the transcription of four cancer-related genes: ADAM metallopeptidase domain 19 (ADAM19), integrin alpha 2 (ITGA2), and fermitin family member 1 (FERMT) 38, 39. Moreover, four serine/threonine (S/T) residues of ETV1 can be phosphorylated by MAPK4 to reduce the ubiquitination degradation of full-length ETV1 (ETV1-FL) 38. Xiao et al. found that the knockout of SRSF3 promotes the skipping of ETV1-FL exon 7 (ETV1-∆E7) in glioma stem-like cells (GSCs). ETV1-∆E7 cannot be phosphorylated by MAPK4 and thus exhibits lower stability. In this way, ETV1-∆E7 inhibits its target gene expression and impairs the growth and sphere formation ability of GSCs 40.

Some kinase activity is also related to SRSF3. Only Iso2 (exon 16 inclusion) of mitogen-activated protein 4 kinase 4 (MAP4K4) can interact and phosphorylate c-Jun N-terminal kinase (JNK) 41. Overexpressed SRSF3 binds to the CU element in exon 16 and promotes the formation of MAP4K4 Iso2, which further activates the JNK signaling pathway 42. JNK signaling pathways widely promote the migration and invasion of cancer cells 43. SRSF3 increases the expression of the EMT-related genes N-cadherin and vimentin, thus promoting the invasion and migration of colorectal cancer (CRC) cells 42.

SRSF3 can also promote migration in TNBC. Cortisol-induced SRSF3 overexpression can promote exon 9 inclusion of GR and subsequently form GRα. In contrast to GR, GRα can respond to glucocorticoid actions 44. Erica et al. found that GRα promotes the transcriptional regulation of receptor for activated C kinase 1 (RACK1), which can further increase the migration of MDA-MB-231 cell lines 45. TNBC lacks effective treatment strategies, but approximately 25% of invasive TNBC is known to be glucocorticoid receptor (GR)-positive 46. SRSF3 might be a novel therapy target for GR-positive TNBC.

Forkhead box transcription factor M1 (FoxM1) is also a transcription factor related to tumor proliferation 47. It has been reported that SRSF3 knockdown promotes FoxM1 exon 9 skipping and downregulates the expression of FoxM1 and its two transcriptional targets PLK1 and Cdc25B, both of which are cell cycle-related proteins. As a result, decreased expression of FoxM1, PLK1 and Cdc25B results in inhibition of the G2/M phase transition. Therefore, it hinders the progression of the cell cycle and promotes the apoptosis of U2OS cells 15.

In addition to the G2/M phase transition, SRSF3 plays an important role of in regulating the progression of the G1/S phase transition in colon cancer cells. Knockdown of SRSF3 can reduce the expression of cyclin D1, cyclin D3, cyclin E1, E2F1 and E2F7, which can impair the G1-to-S-phase progression. In this way, SRSF3 induces G1 cell cycle arrest and apoptosis 48.

Knockdown of SRSF3 causes apoptosis in ovarian cancer cells 49. The oncogene MDM4 can effectively promote the occurrence and proliferation of melanoma 50, 51. Overexpressed SRSF3 binds to MDM4 exon 6, promotes the inclusion of exon 6 and forms normal function of MDM4 (MDM4-L), thereby inhibiting p53-mediated target gene transcription and promoting cell cycle arrest and melanoma cell apoptosis 52. Strikingly, the regulation of exon 6 inclusion is also a key determinant of MDM4 protein abundance in normal tissues and cancer cells. Exon 6 skipping can reduce MDM4 protein abundance 52.

Homeodomain-interacting protein kinase-2 (HIPK2) is also related to the apoptosis of cancer cells, and the deletion of HIPK2 increases the proliferation potential of cancer cells 53-55. In addition, full-length HIPK2 can promote the E3 ligase activity of Siah-1 56. Knocking down SRSF3 leads to the skipping of exon 8 of HIPK2, thus promoting the formation of the Δe8 isoform. Because the Δe8 isoform lacks 27 amino acids that mediate its binding to Siah-1, it cannot participate in protein degradation 48. In this way, depletion of SRSF3 promotes the apoptosis of colon cancer cells.

In addition to these solid tumors, significant progress has been made in the treatment of pediatric B-cell acute lymphoblastic leukemias (B-ALL), but the mortality and recurrence rates are still high in childhood cancer 57. CD19 is a cell surface signaling protein. Because it can recruit intracellular kinases, CD19 is considered to play an indispensable role in promoting B cell proliferation and differentiation 58-61. Elena et al. found that knockdown of SRSF3 resulted in enhanced abundance of the CD19 Δex2 isoform. Consistent with this, in relapsed leukemia, low expression of SRSF3 is correlated with high expression of the CD19Δex2 variant. At present, the effective treatment strategy combats CD19 through the application of T cells expressing chimeric antigen receptors (CAR-T) to improve the cure rate of B-ALL in pediatrics 62, 63. However, because of the existence of alternative splicing, a sequence of CD19Δex2 is missing, and the CAR-T cannot recognize CD19Δex2, which reduces the survival rate of pediatric B-ALL 64. It is worth mentioning that the role of the CD19 Δex2 variant in the differentiation and proliferation of normal B cells is unknown.

### SRSF3 and retained intron (RI)

EIF2B5, a major regulator of translation initiation, has been shown to be a marker protein in various cancers 65, 66. EIF2Bε is the largest subunit of EIF2B5, which has two isoforms, truncated eIF2Bε (65 kDa) and full-length eIF2Bε (80 kDa). Under hypoxic conditions, SRSF3 promotes the intron 12 retention of eIF2Bε and produces a premature termination codon (PTC) that inhibits the overall translation and forms truncated eIF2Bε in head and neck carcinoma (HNC) cells (Table 1). This truncated eIF2Bε lacks the binding region of eIF2Bα and an activating guanine exchange factor (GEF) domain 67. Therefore, it cannot bind eIF2Bα (a subunit of EIF2B5) and guanosine triphosphate (GTP) to initiate the translation process. Finally, truncated eIF2Bε improves the survival rate of HNC cancer cells under prolonged or acute hypoxia 68.

### SRSF3 and alternate terminators (A3SS)

Serine/arginine-rich splicing factor 5 (SRSF5) is another important member of the SR protein family that can promote the proliferation of lung cancer cells 69, 70. SRSF5 has recently been discovered as a novel oncogene that promotes oral squamous cell carcinoma (OSCC) tumor formation and proliferation 71. When alternative splicing occurs at the distal 3' splice site of exon 6, it results in a normal functional long-SRSF5 (SRSF5-L). However, when alternative splicing occurs at the proximal 3' splice site of exon 6, a short fragment (approximately 956 bp) is generated due to the presence of an in-frame stop codon (Table 1). Finally, the short fragment either causes RNA degradation induced by nonsense-mediated mRNA decay (NMD) or produces a SRSF5-L lacking the RS domain 72. In general, SRSF3 is positively correlated with SRSF5 protein expression in OSCC. Moreover, overexpression of SRSF3 promotes the utilization of the distal 3' splice site of SRSF5 exon 6, resulting in an increase in SRSF5-L, thereby promoting OSCC proliferation 71.

### SRSF3 and mutually exclusive exons (MEX)

NudE neurodevelopment protein 1 (NDE1) is a dynein-binding protein that regulates various microtubule-mediated processes, including mitosis 73. Because NDE1 terminal exon 9 and exon 9' are mutually exclusive, there are two isoforms in GSCs: NDE1_KMLL (exon 9 inclusion) and NDE1_SSSC (exon 9' inclusion). NDE1_SSSC exists in normal brain tissue and participate in mitosis. However, NDE1- KMLL has the specific function of promoting the growth of GBM cells in the process of mitotic spindle formation. Knockdown of SRSF3 can promote a transition from NDE1_KMLL to NDE1_SSSC (Table 1) 40.

By alternative splicing, the pyruvate kinase muscle (PKM) gene can produce two isoforms: PKM1 (exon 10 skipping) and PKM2 (exon 9 skipping). In addition, exon 9 and exon 10 are mutually exclusive. Most cancer cells mainly express PKM2 to maintain glycolysis-dominant energy metabolism 74. Three splicing factors, SRSF3, polypyrimidine tract binding protein 1 (PTBP1) and heterogeneous nuclear ribonucleoprotein A1 (hnRNPA1), can bind to exon 10 and promote the inclusion of exon 10, reducing the PKM1/PKM2 ratio (Table 1). Three splicing factors, PTBP1, SRSF3 and hnRNPA1, are highly expressed in colorectal cancer compared with adjacent tissue 75. In addition, this splicing process inhibits the proliferation of colon cancer tumors and alters their metabolic patterns, from glycolysis to oxidative phosphorylation 75.

### SRSF3 regulates various splicing variants

#### SRSF3 alone regulates various splicing variants

Interleukin enhancer binding factor 3 (ILF3), a double stranded RNA binding protein, is the main factor regulating the proliferation of cancer cells 76, 77. SRSF3 binds to different motifs on ILF3 pre-mRNA and produces multiple ILF3 isoforms by exclusion/inclusion of ILF3 exon 18 or by selection of an alternative 3' splice site within exon 18. Among them, ILF3 isoform 1 and isoform 2 can promote cell proliferation, while isoform 5 and isoform 7 suppress tumor cell proliferation and the isoform 7 induces cell apoptosis. Rong et al. confirmed that knocking down SRSF3 attenuated the expression of isoform 1 and isoform 2, and thus inhibited in HeLa and U2OS cell proliferation 78. Meanwhile, when SRSF3 is knocked down, increased ILF3 isoform 5 inhibits the proliferation of HeLa and U2OS cells, and increased ILF3 isoform 7 induces apoptosis (Table 2) 79.

As a tumor suppressor, programmed cell death 4 (PDCD4) is downregulated in most tumors 80. The PDCD4 gene consists of 12 exons, and the cassette exon involved in alternative splicing is located between exons 2 and 3. Under normal conditions, total PDCD4 exists in major isoform of PDCD4 (PDCD4 AS-1), but there is little PDCD4 AS-2 (cassette exon inclusion) expression in the cytoplasm. Knockdown of SRSF3 increases the expression level of PDCD4 AS-2 mRNA in the cytoplasm and its nuclear export in colorectal cancer cells (Table 2) 81. Interestingly, SRSF3 can bind directly to PDCD4 mRNA and mediate translational repression of PDCD4 AS-1 15, 27, 82. Taken together, SRSF3 represses PDCD4 AS-1 mRNA at the translational level, and PDCD4 AS-2 mRNA at the alternative splicing and mRNA export levels, finally promoting the proliferation and migration of colorectal tumors.

Interestingly, SRSF3 can interact with the cellular RNA-binding protein, PCBP2, a protein that binds to internal ribosome entry site (IRES) sequences within the genomic RNAs of certain picornaviruses and is required for viral translation. SRSF3 regulates IRES-induced translation initiation 83.

The regulation of SRSF3 on PDCD4 AS-1 mRNA translation proves that SRSF3 can also play a role in the translation process.

#### SRSF3 collaboratively regulates various splicing variants

HER2 is overexpressed in 20-30% of breast cancer cases. The increase in HER2 expression is related to invasive tumors and adverse prognosis 84. At present, three splicing variants (Δ16 HER2/p100/X5) of full-length HER2 have been identified, and these isomers have different functions and roles in cancer development. Knockdown of HnRNP H1 can increase the expression of the X5 variant (exon 15a inclusion) and oncogenic Δ16 HER2 (exon 16 skipping), thus increasing the trend of breast tumor proliferation. Notably, knockdown of SRSF3 results in the transition from the Δ16 HER2 to p100 variant (intron 15 retention), which inhibits the proliferation of breast cancer cells 85. SRSF3 and HnRNP H1 are the first splicing factors shown to produce HER2 splicing variants with different regulatory functions (Table 2).

## The regulation of SRSF3

SRSF3 is frequently upregulated in many human tumors and therefore plays a role as an oncogene in various cancer processes. Cells maintain stable expression of SRSF3 through the splicing mechanism of SRSF3. However, because of its carcinogenic effect, SRSF3 must be strictly monitored (Table 3).

### Splicing regulation of SRSF3

SRSF3 has two splicing forms: the full-length normal form (lacking exon 4, Iso1) and the splicing isoform (containing exon 4, Iso2). Iso2 contains a preterminated codon, which is degraded by NMD to avoid cell toxicity, or it encodes a nonfunctional truncated SRSF3 protein that lacks the RS domain at the C-terminus 86 (Figure 1). Hassan et al. found that SRSF3 can regulate alternative splicing of its own mRNA. Overexpression of SRSF3 attenuates the production of the exon 4-deleted transcript and activates the production of exon 4-containing transcripts 86.

#### SRSF1

Serine/arginine-rich splicing factor 1 (SRSF1, ASF/SF2) was the first member of the SR protein family to be found. As a cancer-promoting factor, SRSF1 can promote the proliferation and EMT of breast epithelial cells 68. SRSF1 has been proven to antagonize the self-splicing effect of SRSF3 exon 4. Overexpressed SRSF3 promotes the recognition of exon 4, whereas SRSF1 inhibits the recognition and antagonizes the inclusion of SRSF3 exon 4, thus producing full-length SRSF3 (Iso1) 17, 86.

#### HnRNP L

Nuclear heterogeneous ribonucleoprotein L (HnRNP L) is a selective pre-RNA splicing factor that maintains translation and RNA stability 36, 87-89. There is a positive correlation between HnRNP L and SRSF3 in OSCC tissues 71, 90. It has been reported that HnRNP L can promote splicing of SRSF3 exon 4 and then promote the proliferation of OSCC cells 90. This suggests that HnRNP L may be a potential carcinogenic target. Therefore, in OSCC, SRSF3 can be spliced by HnRNP L or used as a splicing factor to act on SRSF5 71.

#### SLU7

SLU7, as a key splicing factor of RNA precursors, has an effect on inhibiting the development of HCC and maintaining the homeostasis of normal hepatocytes 91. Moreover, the downregulation of SLU7 can damage liver glucometabolic and lipid metabolism disorders, and increase the risk of cancer 91. Jiménez M et al. also found that deletion of SLU7 can promote the production of truncated SRSF3-Iso2 in a variety of different cancer cell lines. SLU7 knockdown not only increases the abnormal expression of truncated SRSF3 but also leads to the altered proliferation and abnormal metabolism of tumor cells 92.

#### PTBPs and RBM4

PTBPs (PTBP1 and PTBP2) can bind to the CU element of SRSF3 exon 4, impairing the automatic splicing regulation of SRSF3 and promoting SRSF3 exon 4 skipping 93. Intriguingly, RNA-binding motif protein 4 (RBM4) is an antagonist of PTBP that can bind to intronic CU elements and promote the inclusion of SRSF3 exon 4, decrease full-length SRSF3 expression and inhibit the proliferation of CRC cells 94. Overall, PTBP1 can promote the skipping of SRSF3 exon 4, and increased SRSF3 enhances the production of MAP4K4 Iso2 and Iso5 in HCT-8 and HCT-116 cells. When the expression of RBM4 is higher than that of PTBP1, RBM4 promotes the inclusion of SRSF3 exon 4 and the production of the MAP4K4 Iso1 variant in Colo205 cells 42.

#### circSMARCA5

Imbalances in the expression of circular RNAs (circRNAs) have been emphasized in several types of cancers 95-97. Without splicing, circSMARCA5 is a novel tumor suppressor that inhibits the migration of glioblastoma multiforme (GBM) cells 98. Barbagallo et al. have found that the overexpression of circSMARCA5 increased the inclusion of SRSF3 exon 4 (Iso2) and that the downregulation of circSMARCA5 significantly increased the skipping of SRSF3 exon 4 (Iso1) in the process of splicing SRSF3 by SRSF1. In addition, the ratio of SRSF3 Iso2/ Iso1 is positively correlated with circSMARCA5 expression 98.

### Ubiquitination

DARPP-32, also named dopamine and cAMP-regulated phosphoprotein (Mr 32000) 99. DARPP-32 is overexpressed in 2/3 early stage gastric cancer patients and can increase the survival rate, drug resistance and invasive activity of gastric cancer cells 100-102. DARPP-32 can enhance the SRSF3 protein expression. The reason is that DARPP-32 can significantly reduce the ubiquitination of SRSF3 in digoxin-induced AGS cells. Conversely, the elimination of endogenous DARPP-32 in MKN-45 cells significantly increases the ubiquitination of SRSF3. DARPP-32 can enhance the stability of SRSF3 and regulate SRSF3-induced CD44 splicing, thus promoting CD44E expression and gastric growth 103. However, the detailed ubiquitination mechanism is still elusive.

### Neddylation

Deepak et al. found that the expression of SRSF3 decreased in early human liver diseases, including nonalcoholic fatty liver disease (NAFLD), nonalcoholic steatohepatitis (NASH), or cirrhosis 104. Neddylation is a posttranslational modification that is similar to ubiquitination 105. NEDD8 is a member of the ubiquitin-like protein family and covalently binds to Lys residues of substrates 106. Under oxidative stress induced by palmitic acid, NEDD8 promotes the neddylation of SRSF3 at 11 lysine sites, resulting in SRSF3 degradation in liver diseases. When lysine 11 mutation (K11R) occurs, SRSF3 neddylation is reduced, protecting SRSF3 from degradation and reducing the risk of liver disease and further progression to HCC 104. SRSF3 is regulated in a neddylation-dependent manner in the liver, which provides an effective therapeutic strategy for suppressing liver diseases and hepatocellular carcinogenesis.

### Phosphorylation

Similar to SR proteins, SRSF3 is mainly located in the nucleus. In the absence of splicing, the SRSF3 protein mainly aggregates into irregular granular nuclei and becomes a typical structure in the nucleus, which is called a "speckle" 107.

SRSF3 is also a nuclear shuttle protein. Its shuttling property is related to the phosphorylation of the RS domain 108. When serine (residue 105 relative to the C-terminus) in the RS domain is phosphorylated by serine-arginine protein kinase 2 (SRPK2), the SR protein can be released from the speckles in the nucleus. Then, the N-terminal RRM domain specifically binds to the pre-mRNA to complete splicing with other splicing factors 109. Therefore, the phosphorylation of SR protein is very important to the shuttling property of SR protein. SRSF3 is hypophosphorylated in cell homeostasis. Enhanced phosphorylation of SRSF3 promotes the occurrence of splicing events mediated by carcinogenic SRSF3 and the generation of GBM tumors 40. In addition, Wang et al. found that hypothalamic gonadotropin-releasing hormone (GnRH) has normal reproductive function and can induce phosphorylation of SRSF3 in a time-dependent way 110.

## Conclusions

SRSF3 is upregulated in many kinds of tumors and functions as a proto-oncogene. However, SRSF3 expression is decreased in >50% of human HCCs 111. Specific loss of SRSF3 in hepatocytes impairs the differentiation and metabolism of hepatocytes in early adulthood 112. Hepatocyte-specific SRSF3 knockout mice are at risk of liver fibrosis, while SRSF3 can protect mice from CCL4-induced fibrosis and carcinogenesis by inhibiting the inclusion of EDA fibronectin 1 exon 111. In addition, deletion of SRSF3 promotes the skipping of Git2 exon 15/Slk exon 13, the inclusion of Myo1B exon 23/Ctnnd1 exon 18, and the development of HCC metastasis with aging 113.

In alternative splicing, most of the isomers produced by SRSF3 have different phenotypes, which are manifested in promoting and suppressing cancer. SRSF3 can regulate the cell cycle, apoptosis, cell metastasis and proliferation according to different splicing variations (Figure 2B).

At present, although it is known that SRSF3 can splice related proteins, the mechanism by which SRSF3 splices related proteins are still unclear. In addition, the splicing role of SRSF3 in tumorigenesis and its clear molecular mechanism deserve further investigation. Elucidation of the molecular mechanism of abnormal splicing of SRSF3 caused by tumorigenesis could be of great significance in clarifying the occurrence and development of tumors.

## Figures and Tables

**Table 1:** SRSF3 regulates splicing variants

Classification	SRSF3	Splicing proteins	Mechanisms	Isoform	Tumor types	Cell lines	Molecular functions	Reference
**Exon-skip (ES)**	Promote	CCDC50	Exon 6 skipping	CCDC50S	HCC	HepG2 cells Bel-7402 cells Bel-7404 cells	Increase the proliferation and migration	28
		CD44	Exons12-14 skipping	CD44E	Gastric cancer	MKN-45 cells	Increase the gastric tumorigenesis	32, 114
		PAR3	Exon 12 skipping	Δ12PAR3 isoform	TNBC	MDA-B231 cells	Increase metastasis and proliferation	37
		NUMB	Exon 12 skipping	Δ12NUMB isoform	TNBC	MDA-MB231 cells	Increase metastasis and proliferation	37
		Git2	Exon 15 skipping	—	HCC	HKO mice	Mediate EMT in mice	111
		Slk	Exon 13 skipping	—	HCC	HKO mice	Mediate EMT in mice	111
	Inhibit	ETV1	Exon 7 skipping	ETV1-∆E7	GBM	GSC83 cells	Impair the growth and sphere formation ability of GSCs	40
		MAP4K4	Exon16 skipping	MAP4K4 Iso2	CRC	HCT-8 cells	Promote the migration and invasion of CRC cell	42
		GR	Exon 9 skipping	GRα	TNBC	MDA-MB-231 cells	Promote transcriptional regulation and cell migration	45
		FoxM1	Exon 9 skipping	—	Osteosarcoma	U2OS cells	Affects G2/M arrest, growth retardation and apoptosis	15
		MDM4	Exon 6 skipping	MDM4-S	Melanoma	A375 cells	Promote cell cycle arrest and melanoma cell apoptosis	52
		HIPK2	Exon 8 skipping	HIPK2 ΔE8	Colon cancer	HCT116 cells	Decrease cell cycle progression from G1 to S phase	48
		CD19	Exon 2 skipping	CD19 ΔEx2	B-ALL	697 cells; NALM-6 cells	Increase the abundance of CD19 Δex2 variant	114
		Myo1B	Exon23 skipping	—	HCC	HKO mice	Mediate EMT in mice	111
		Ctnnd1	Exon18 skipping	—	HCC	HKO mice	Mediate EMT in mice	111
**Retained intron (RI)**	Overexpression	eIF2Bϵ	Intron retention	Truncated eIF2Bε	HNC	Hypoxic SQ20B cells	Increase HNC cancer cells growth	115
**Alternate terminators(A3SS)**	Overexpression	SRSF5	Exon 6 inclusion	SRSF5-S	OSCC	CAL 27 cells	Promote OSCC proliferation	71
**Mutually exclusive exons** **(MEX)**	Overexpression	NDE1	Exon 9 inclusion Exon 9' inclusion	NDE1_KMLL NDE1_SSSC	GBM	GSC83 cells	Promote the growth of GBM cells Inhibit the growth of GBM cells	40
	Knockdown Overexpression	PK	Exon 10 skipping Exon 9 skipping	PKM1 PKM2	Colon cancer	DLD-1 cells	Inhibits the proliferation and alters metabolic patterns of colon cancer	75

**Table 2:** SRSF3 regulates various splicing variants

Coordination	Splicing proteins	Mechanisms	Isoform	Tumor types	Cell lines	Molecular functions	Reference
—	ILF3	Exon 18 skipping Exon 18 inclusion	ILF3 isoform -1 ILF3 isoform -2	Osteosarcoma	U2OS cells; MEF3T3 cells	Promote cell proliferation and transformation	79
—	PDCD4	Cassette exon skipping Cassette exon inclusion	PDCD4 AS-1 PDCD4 AS-2	CRC	SW480 cells	Reduce PDCD4 protein expression	81
hnRNP H1	HER2	Exon 16 skipping Intron 15 retention Exon 15a inclusion	Δ16 HER2 p100 X5	Breast cancer	SKBR3 cells	Inhibit the proliferation of breast cancer	85

**Table 3:** The regulation of SRSF3 in tumor

Regulation	Splicing proteins	Mechanisms	Isoform	Tumor types	Cell lines	Molecular functions	Reference
Splicing regulation	SRSF1	Exon 4 skipping	SRSF3(Iso1)	Osteosarcoma	U2OS cells HeLa cells	Inhibit the expression in cancer cells	86
	HnRNP L	Exon 4 skipping	SRSF3(Iso1)	OSCC	CAL 27 cells	Inhibit the proliferation of OSCC cells	71
	SLU7	Exon 4 inclusion	SRSF3(Iso2)	Hepatocytes	AAV-SLU7 cells	Maintain liver homeostasis	91
	PTBPs	Exon 4 skipping	SRSF3(Iso1)	CRC	HepG2 cells	Decrease the proliferation and migration	42
	RBM4	Exon 4 inclusion	SRSF3(Iso2)	CRC	HepG2 cells	Decrease the proliferation and migration	42
	circSMARCA5	Exon 4 inclusion	SRSF3(Iso2)	GBM	U87MG cells	Inhibit GBM migration	98
Other regulation	Ubiquitination	DARPP-32	SRSF3 ↑	Gastric cancer	AGS cells	Increase the gastric tumorigenesis	103
	Neddylation	NEDD8	SRSF3 ↓	HCC	HepG2 cells	Increase the risk of liver disease	104
	Phosphorylation	SRPK2	SRSF3 ↑	GBM	HEK293cells	Promote the generation of tumors	109

## Abbreviations

SRSF3: Serine/arginine-rich splicing factor 3
HCC: Hepatocellular Carcinoma
TNBC: Triple- negative breast cancer
GSCs: Glioma stem-like cells
OSCC: Oral squamous cell carcinoma
B-ALL: B-cell acute lymphoblastic leukemias
CRC: Colorectal cancer
HNC: Head and neck carcinoma
GBM: Glioblastoma multiforme
ETV1: Transcription factor ETS variant 1
MAP4K4: Mitogen-activated protein 4 kinase 4
GR: Glucocorticoid receptor
FoxM1: Forkhead box transcription factor M1
HIPK2: Homeodomain-interacting protein kinase-2
SRSF5: Serine/arginine-rich splicing factor 5
NDE1: NudE neurodevelopment protein 1
PKM: Pyruvate kinase muscle
ILF3: Interleukin enhancer binding factor 3
PDCD4: Programmed cell death 4
SRSF1: Serine/arginine-rich splicing factor 1
HnRNP L: Nuclear heterogeneous ribonucleoprotein L
PTBPs: Polypyrimidine tract binding proteins
RBM4: RNA-binding motif protein 4
SRPK2: Serine-arginine protein kinase 2
